# Supplementary material for: Patterns of Intron Gain and Loss in Fungi
Source: PLoS Biol. 2004 Nov 30;2(12):e422. doi: 10.1371/journal.pbio.0020422 (PMC532390; doi:10.1371/journal.pbio.0020422)
Supplement: Table S1 — Also available at http://genes.mit.edu/NielsenEtAl/. (4.3 MB ZIP). [file pbio.0020422.st001.zip › NielsenEtAl/html/142.html]

AN0818.1.NCU02619.1.MG01205.1.FG00665.1


```
 CLUSTAL W (1.82) Multiple Sequence Alignments - Introns Inserted


Sequence 1: NCU02619.1	737 aa
Sequence 2: FG00665.1	767 aa
Sequence 3: MG01205.1	739 aa
Sequence 4: AN0818.1	714 aa
Alignment Length: 788 aa
Number Identitical Residues: 291 aa
Alignment Score (without introns) 15400


MG01205.1 	MDPNATTPI-PHPP-----ELASTAEKRLIEAENGEPAAKRVRLDE------DGGENARD
NCU02619.1	MESQETAKR-PIEP------------NGAVAAQDAEPATKRVKLDD------APVPQIQE
FG00665.1 	MNEQPDAPMGDVSPGHEHAELIADASNIETSTDDGERASKRVKMNDSIPAESVPGEGLHD
AN0818.1  	MEGTAQNLDVEVPK----------QDLPNGTDSTGEPPLKKVRLEEP-------------
          	*:                       .      . .* . *:*::::.             

MG01205.1 	SA--------VKGDAANGQEHITPAASEIKPDNRVKGQAPIKAE2YLVEVIHNNGRDARD
NCU02619.1	EPSQPQ----PQPQPQPQTEDEKPTEQRQDDRDKRRGIAPIKKE2YLVVPPSQVAKTA-E
FG00665.1 	APASKENGNVTNGDKENLTEEQNPDSKPEHVDGRIKGIAPIKKE2YLVDLSTFKDIST-D
AN0818.1  	--------------ASDQQNGQAPP--------RLKGVAPIKAE2FLIPKPKQ-GQAA--
          	                   :   *         : :* **** * :*:         :  

MG01205.1 	EGPDDDAAEGRTASEPSG------------KVARKGKKGQNQARDFGRSADSIRLCNSRM
NCU02619.1	VV-DDDAAEGRTAPGAEG-----AAGK---DGKKKRPKGQNKEREFGIFADAQRLCNSVA
FG00665.1 	VVNDDDAAEARGTADNEGQGDNRKAGKGKKDKKDKKKKGQNTERDFGKFDDAFRLCNSRA
AN0818.1  	VSSTDDAAEAAAYKDREDE----------KKGKKKKTTGQNKNRTFGRSQDSKGLCASRV
          	    *****.      ...          ..   *  .***  * **   *:  ** *  

MG01205.1 	LSDEFSPKHCSFGDRCNLSHDLRKYLAEGRRPDLETFGGKCPIFEVHGWCNAGWKCLFVR
NCU02619.1	WTPEFSPRHCKHGERCNALHDIRKYLKEGRRPDLNVFGGKCPVWETHGKCSSGWRCLFVE
FG00665.1 	FYPEFSPRECRFGDRCNLSHDLRKYLEEGRRGDVETFEGKCPVFEAHGHCPAGWKCRFVK
AN0818.1  	YSPEFSPAECPFGDKCRFEHDLRTYLKEHKREDLNTFNGVCPVWSARGKCDAGWKCRFVG
          	   **** .* .*::*.  **:*.** * :* *::.* * **::..:* * :**:* ** 

MG01205.1 	SHMEEVEREDGRKELVLLRDLTRSK------KPEGEASEDGPYGVVNTVASVDKHRMGRS
NCU02619.1	SHMKEIEHEDGRKELVLTEDPTKIKPEAAPLVPPEEESMDARAGVYNVVAPAVKLALSRK
FG00665.1 	SHMKEIEHEDGRKELVLISTSANGGENGE---PADEGSEEKRPAIYNVVGIDKKIALNRK
AN0818.1  	SHSIERVTEDGRKELVLVEDEERRK------KAQPVFPSAAEDGTVNTTSPADKIALAKR
          	**  *   *********     .         .    .     .  *...   *  : : 

MG01205.1 	KVDLSRSVEYSKWMDQYTNLVKQLHN-------ARVDEKKREEELGELRATFVEPPFKPS
NCU02619.1	KIQHEKSDQYLKWMAKDSELARIHYH-------KQKDD---DDVAKDYAAQYVEPPLKPS
FG00665.1 	RTDFTQSEQYIGWLNKEAKLSEEFMN-------RRKNQS--DEGIEDLRARYIDPPFKPS
AN0818.1  	QRPTPRADAYSDWLDLTSKELEKYFHGGQQNKDAHPDTAQDKEEKEENRATYTEAPFMPS
          	:    ::  *  *:   ::  .   :....... : :  . .:   :  * : :.*: **

MG01205.1 	EKRRLYFGRETPVLAPLTTQGNLPFRRLCVDLGAQGTYSEMAMAKPIVQGQQSEWALMKA
NCU02619.1	EKRRIYFGRETPVLAPLTTQGNLPFRRLCVELGAEITYSEMALGLPLLQGQKADWTLMRA
FG00665.1 	EKRRLYFGAETPTLAPLTTQGNLPFRRLCVELGAQLTYSEMVLSMPLIQGTKADWTLVKA
AN0818.1  	EKRRLYFGPETPALAPLTTQGNLPFRRLCVELGAQLTYSEMALSMPLIQGHKPEWALMRA
          	****:*** ***.*****************:***: *****.:. *::** :.:*:*::*

MG01205.1 	HESETRPPTLSPETLASNSPIVKVYDNSRDLRFGAQISASSHPLAIKAAECLTTFLPHLR
NCU02619.1	HESEITPPRYTP-----TGIVDPAYDNSKDLKFGVQITAHAPWIAIKSAETLARYLPHLR
FG00665.1 	HESEITPPKFNPG----SVPIFDNYNHSKDIKFGAQISGNNHWVVTKAADVLNRFCPHLR
AN0818.1  	HETEALPPTVSAR-----ASVVQDYDNSKDMKFGAQIAGNKYRWVMKATEVLSSLTPNLR
          	**:*  **  ..        :   *::*:*::**.**:.     . *::: *    *:**

MG01205.1 	LIDLNCGCPIDMIYKAGAGSALLEQHNKIERIIRGMNAVSGEVPITCKLRIGVKENHPLA
NCU02619.1	VIDLNCGCPIDMVYKSGAGSALLDAPSKLERMIRGMNTVSGEVPVTAKIRMGVRDNHLTA
FG00665.1 	LIDLNCGCPIDMVFKSGGGSALLENSGKLERMIRGMNAVSGEVPITAKIRTGIRNSRPTA
AN0818.1  	VIDLNCGCPIDLLYREGSGSALLDAPSKLEKMLRGMNAVSEQIPITVKIRTGTRDNTPNA
          	:**********:::: *.*****:  .*:*:::****:** ::*:* *:* * ::.   *

MG01205.1 	KKNIERAVFGSPDFRNQLGAP-GCAAITLHGRTRQQRYKKPADWGYIAECAALIQSYRDK
NCU02619.1	QKLVERLALGAPDIRAVSGAP-GCAAVTLHGRTRQQRYTKAADWSYIAECAALVKSFNEK
FG00665.1 	TQLIGKLAFGAREHRERLGAP-GCAALTLHGRSREQRYTKKADWGYISECAALIKTYNKE
AN0818.1  	QKLVERLILGGHEASMLNCGPSGVAAITLHGRSRQQRYTREANWEYISETAALIKRLNEK
          	 : : :  :*. :      .*:* **:*****:*:***.: *:* **:* ***::  ..:

MG01205.1 	RDELTDTVREPDASTLAPS--SEVFFLGNGDCYSHADYYKAIDESHVDTVMIGRGALIKP
NCU02619.1	ADSLQDTIREADERSLPNG--GRMYFVGNGDCYSHVDYFNHVDNAKVDSVMVGRGAIIKP
FG00665.1 	KDSLTDTIAEPDASSLPNAKDGRMYFLGNGDCYSHVEYYEHIEKARVDTVMIGRGALIKP
AN0818.1  	SDEVTDTIREPEERMRPNG--GKTWFLGNGDCYSHVDYEDHIKNAKVDSVMVGRGALIKP
          	 *.: **: *.:    . .  .. :*:********.:* . :.:::**:**:****:***

MG01205.1 	WVFEEIAAGQYLDKSSTERLGYIERFCRYGMEAWGADEMGIGTTRRFLLEYLSFAYRYVP
NCU02619.1	WIFEEISAGQYLDKSATERLAYVEKFAKYGMEAWGSDELGLNYTRRFLLEFLSFFHRYVP
FG00665.1 	WLFEEIEKGQYLDKSSSERLRYIEKFVRYGLDAWGSDELGIGFTRRFLLEWLSFAHRYVP
AN0818.1  	WLFEEIQAGQYLDKSASERLAYVEKFARYGMETWGSDEYGIGITRRFLLEWLSFACRYVP
          	*:****  *******::*** *:*:* :**:::**:** *:. *******:***  ****

MG01205.1 	IGLLERLPPAINDRPPAYRGRDDLETLMASDNYRDWIKIS2EMFLGPAHEGFKFQPKHKS
NCU02619.1	IGLLEYLPPAMNDRPPAYKGRNELETLLASKNYLDWIKIS2EMFLGPAPPNFKFQPKHKS
FG00665.1 	IGLLERLPPDLNDRPPAYEGRDEMETLMASSNFRDWIKIS2EMFLGPVHPTFKFQPKHKS
AN0818.1  	IGLLEYLPPKINDRPPYWRGRNDMETLMGSHDYRDWIKIS2EMFLGPAHKDFKFEPKHKS
          	***** *** :***** :.**:::***:.* :: ****** ******.   ***:*****

MG01205.1 	NSYE-MEAEG
NCU02619.1	NAYENIEAEG
FG00665.1 	NSYE---AEG
AN0818.1  	NSYD---TEG
          	*:*:   :**
```
